# Consensus properties for the deep coalescence problem and their application for scalable tree search

**DOI:** 10.1186/1471-2105-13-S10-S12

**Published:** 2012-06-25

**Authors:** Harris T Lin, J Gordon Burleigh, Oliver Eulenstein

**Affiliations:** 1 Department of Computer Science, Iowa State University, Ames, IA, USA; 2 National Evolutionary Synthesis Center, Durham, NC, USA; University of Florida, Gainesville, FL, USA

## Abstract

**Background:**

To infer a species phylogeny from unlinked genes, phylogenetic inference methods must confront the biological processes that create incongruence between gene trees and the species phylogeny. Intra-specific gene variation in ancestral species can result in deep coalescence, also known as incomplete lineage sorting, which creates incongruence between gene trees and the species tree. One approach to account for deep coalescence in phylogenetic analyses is the deep coalescence problem, which takes a collection of gene trees and seeks the species tree that implies the fewest deep coalescence events. Although this approach is promising for phylogenetics, the consensus properties of this problem are mostly unknown and analyses of large data sets may be computationally prohibitive.

**Results:**

We prove that the deep coalescence consensus tree problem satisfies the highly desirable Pareto property for clusters (clades). That is, in all instances, each cluster that is present in all of the input gene trees, called a consensus cluster, will also be found in every optimal solution. Moreover, we introduce a new divide and conquer method for the deep coalescence problem based on the Pareto property. This method refines the strict consensus of the input gene trees, thereby, in practice, often greatly reducing the complexity of the tree search and guaranteeing that the estimated species tree will satisfy the Pareto property.

**Conclusions:**

Analyses of both simulated and empirical data sets demonstrate that the divide and conquer method can greatly improve upon the speed of heuristics that do not consider the Pareto consensus property, while also guaranteeing that the proposed solution fulfills the Pareto property. The divide and conquer method extends the utility of the deep coalescence problem to data sets with enormous numbers of taxa.

## Introduction

The rapidly growing abundance of genomic sequence data has revealed extensive incongruence among gene trees (e.g., [1,2]) that may be caused by processes such as deep coalescence (incomplete lineage sorting), gene duplication and loss, or lateral gene transfer (see [3-5]). In these cases, phylogenetic methods must account for and explain the patterns of variation among gene tree topologies, rather than simply assuming the gene tree topology reflects the relationships among species. In particular, there has been much recent interest in phylogenetic methods that account for deep coalescence, which may occur in any sexually reproducing organisms (e.g., [6-8]). One such approach is the deep coalescence problem, which, given a collection of gene trees, seeks a species tree that minimizes the number of deep coalescence events [4,9]. Although the deep coalescence problem is NP-hard [10], recent algorithmic advances enable scientists to solve instances with a small number of taxa [11,12] and efficiently compute heuristic solutions for data sets with slightly more species [13]. Still, the heuristics are based on generic local tree search strategies with no performance guarantees, and they cannot handle enormous data sets. In this study, we prove that the deep coalescence problem satisfies the Pareto consensus property. We then describe a new divide and conquer approach, based on the Pareto property, that, in practice, can greatly extend the utility of existing heuristics while guaranteeing that the inferred species tree also has the Pareto property with respect to the input gene trees.

### Related work

The deep coalescence problem is an example of a supertree problems, in which input trees with taxonomic overlap are combined to build a species tree that includes all of the taxa found in the input trees (see [14]). In fact, it is among the few supertree methods that use a biologically based optimality criterion. One way of evaluating supertree methods is by characterizing their consensus properties (e.g., [15,16]). The consensus tree problem is the special case of the supertree problem in which all the input trees contain the same taxa. Since all supertree problems generally seek to retain phylogenetic information from the input trees, one of the most desirable consensus properties is the Pareto property. A consensus tree problem satisfies the Pareto property on clusters (or triplets, quartets, etc.) if every cluster (or triplet, quartet, etc.) that is present in every input tree appears in the consensus tree [15-17]. Many supertree problems satisfy the Pareto property for clusters in the consensus setting [15,16]. However, this has not been shown for the deep coalescence problem.

### Our contributions

We prove that the deep coalescence consensus tree problem satisfies the Pareto property for clusters. This result provides useful guidance for the species tree search. Instead of evaluating all possible species trees, to find the optimal solution we need only to examine trees that satisfy the Pareto property on clusters. These trees will all be refinements of the strict consensus of the gene trees. Furthermore, the Pareto property allow us to show that the problem can be divided into smaller independent subproblems based on the strict consensus tree. We apply this property and describe a new divide and conquer method, and our experiments demonstrate that this method can greatly improve the speed of deep coalescence tree heuristics, potentially enabling efficient and effective estimates from inputs with several thousands of taxa. Future work will exploit the independence of the subproblems and solve these on parallel machines, which should result in even larger and more accurate solutions.

## Methods

### Basic definitions, notations, and preliminaries

In this section we introduce basic definitions and notations and then define preliminaries required for this work. For brevity some proofs are omitted in the text but available in Additional file 1.

A *graph G *is an ordered pair (*V, E*) consisting of a non-empty set *V *of *nodes *and a set *E *of *edges*. We denote the set of nodes and edges of *G *by *V*(*G*) and *E*(*G*), respectively. If *e *= {*u, v*} is an edge of a graph *G*, then *e *is said to be *incident *with *u *and *v*. If *v *is a node of a graph *G*, then the *degree *of *v *in *G *is the number of edges in *G *that are incident with *v*.

A *tree T *is a connected graph with no cycles. *T *is *rooted *if it has exactly one distinguished node of degree one, called the *root*, and we denote it by Ro(T). The unique edge incident with Ro(T) is called the *root edge*.

Let *T *be a rooted tree. We define ≤*_T _*to be the partial order on *V *(*T*) where *x *≤*_T_ y *if *y *is a node on the path between Ro(T) and *x*. If *x *≤*_T_ y *we call *x *a *descendant *of *y*, and *y *an *ancestor *of *x*. We also define *x *<*_T_ y *if *x *≤*_T_ y *and *x *≠ *y*, in this case we call *x *a *proper descendant *of *y*, and *y *a *proper ancestor *of *x*. The set of minima under ≤*_T _*is denoted by Le(T) and its elements are called *leaves*. A node is *internal *if it is not a leaf. The set of all internal nodes of *T *is denoted by *I*(*T*). Further, we will frequently refer to the subset of *I*(*T*) whose degree is two, and we denote this by *I*_2_(*T*).

Let $X\subseteq Le\left(T\right)$, we write $\bar{X}$ to denote the *leaf complement *of *X *when the tree *T *is clear from the context, where $\bar{X}=\text{ L}e\left(T\right)\backslash X$.

If {*x, y*} ∈ *E*(*T*) and *x *<*_T_ y *then we call *y *the *parent *of *x *denoted by ${Pa}_{T}\left(x\right)$ and we call *x *a *child *of *y*. The set of all children of *y *is denoted by ${Ch}_{T}\left(y\right)$. If two nodes in *T *have the same parent, they are called *siblings*. The *least common ancestor *(LCA) of a non-empty subset *X *⊆ *V*(*T*), denoted as *lca_T_*(*X*), is the unique smallest upper bound of *X *under ≤*_T_*.

If *e *∈ *E*(*T*), we define *T*/*e *to be the tree obtained from *T *by identifying the ends of *e *and then deleting *e*. *T*/*e *is said to be obtained from *T *by *contracting e*. If *v *is a vertex of *T *with degree one or two, and *e *is an edge incident with *v*, the tree *T*/*e *is said to be obtained from *T *by *suppressing v*.

**Figure 1:**
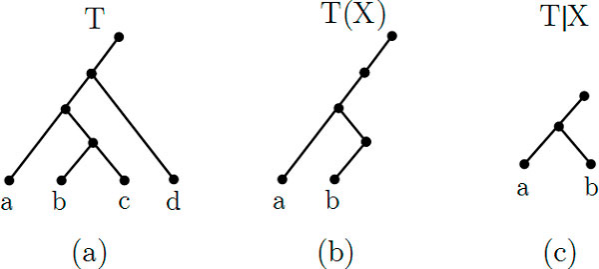
**Examples of tree definitions**. (a) A rooted tree *T *with four leaves {*a, b, c, d*}. (b) The subtree of *T *induced by *X *where *X *= {*a, b*}. (c) The restricted subtree of *T *induced by *X*.

Examples of the following definitions are shown in Figure 1. Let *X *⊆ *V*(*T*), the *subtree *of *T induced *by *X*, denoted *T*(*X*), is the minimal connected subtree of *T *that contains Ro(T) and *X*. The *restricted subtree *of *T *induced by *X*, denoted as *T*|*X*, is the tree obtained from *T*(*X*) by suppressing all nodes with degree two. The *subtree *of *T *rooted above node *v *∈ *V *(*T*), denoted as *T_v_*, is the restricted subtree induced by {*u *∈ *V *(*T*): *u *≤*_T_ v*}.

*T *is *binary *if every node has degree one or three. Throughout this paper, the term tree refers to a rooted binary tree unless otherwise stated. Also, the subscript of a notation may be omitted when it is clear from the context.

#### Deep coalescence

**Figure 2:**
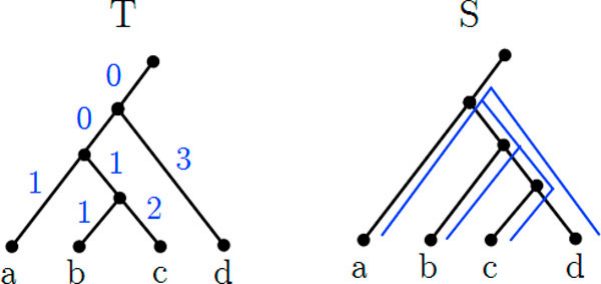
**Example of deep coalescence cost definition**. Example showing the deep coalescence cost from *T *to *S*. Each edge of *T *is accompanied by its cost, and its corresponding path is shown on *S*.

We define the *deep coalescence *cost function as demonstrated in Figure 2. Note that our definition of the deep coalescence cost given in Def. 3, is somewhat different, but for our purposes equivalent, to its original definition also termed *extra lineage *given in [4]. The relationship between both definitions is shown in Additional file 1.

Throughout this section we assume *T *and *S *are trees over the same leaf set.

**Definition 1 **(Path length). *Suppose x *≤*_T_ y, the *path length *from x to y, denoted pl_T_*(*x, y*), *is the number of edges in the path from x to y*. *Further, let *$X\subseteq Y\subseteq Le\left(T\right)$, *we extend the path length function by pl_T_*(*X,Y*) ≜ *pl_T_*(*lca_T_*(*X*), *lca_T_*(*Y*)).

**Definition 2 **(LCA mapping). *Let v *∈ *V*(*T*), *the *LCA mapping *of v in S, denoted M_T⊳S_*(*v*), *is defined by *$M_{T⊳S}\left(v\right)\;≜lca_{S}\left(Le\left(T_{v}\right)\right)$.

**Definition 3 **(Deep coalescence). *The *deep coalescence cost *from T *to *S, denoted DC*(*T, S*), *is*

$$DC\left(T,\;S\right)≜\underset{\begin{array}{c} \left\{u,v\right\}\in E\left(T\right)\\ u<v \end{array}}{\sum }pl_{S}\left(M_{T⊳S}\left(u\right),\;M_{T⊳S}\left(v\right)\right)$$

Using the extended path lengths, the deep coalescence cost can be equivalently expressed as

$$DC\left(T,\;S\right)=\underset{\begin{array}{c} \left\{u,v\right\}\in E\left(T\right)\\ u<v \end{array}}{\sum }pl_{S}\left(Le\left(T_{u}\right),\;Le\left(T_{v}\right)\right)$$

#### Consensus tree

**Definition 4 **(*Consensus tree problem*). *Let *f:Tx×Tx→R*be a cost function where X is a leaf set and *Tx*is the set of all trees over X. A consensus tree problem based on f is defined as follows*.

*Instance: A tuple of n trees *(*T*_1_,...,*T_n_*) *over X*

*Find: The set of all trees that have the minimum aggregated cost with respect to f. Formally*,

$$\underset{S\in T_{X}}{\mathrm{argmin}}\left(\overset{n}{\underset{i=1}{\sum }}f\left(T_{i},S\right)\right)$$

*This set is also called the *solutions *for the consensus tree instance*.

**Definition 5 **(Deep coalescence consensus tree problem). *We define the *deep coalescence consensus tree problem *to be the consensus tree problem based on the deep coalescence cost function*.

#### Cluster and Pareto

**Definition 6 **(Cluster). *Let T be a tree, the clusters induced by T, denoted*, $Cl\left(T\right)$*, is *$Cl\left(T\right)≜\left\{Le\left(T_{v}\right):v\in V\left(T\right)\right\}$. *Further, $X\in Cl\left(T\right)$ is called a trivial cluster if *$X=Le\left(T\right)$ or |*X*| = 1, *it is called non-trivial otherwise*. *Let *$Y\subseteq Le\left(T\right)$, we say that *T *contains *(cluster) Y *if $Y\in Cl\left(T\right)$.

**Definition 7 **(Pareto on clusters). *Let P be a consensus tree problem based on some cost function. We say that P is Pareto on clusters if: for all instances I *= (*T*_1_,...,*T_n_*) *of P, for all solutions S of I, we have *$\cap_{i=1}^{n}\text{C1}\left(T_{i}\right)\subseteq \text{C1}\left(S\right)$.

### Theorem overview

We wish to show that the deep coalescence consensus tree problem is Pareto on clusters. We describe a high level structure of the proof in this section and provide necessary supporting lemmata in the next section. The proof proceeds by contradiction, assuming that the deep coalescence consensus tree problem is *not *Pareto on clusters. By Def. 7, the assumption implies that there exists an instance *I *= (*T*_1_,...,*T_n_*), a solution *S *for *I*, and a cluster $X\subseteq Le\left(S\right)$ where $X\in \cap_{i=1}^{n}Cl\left(T_{i}\right)$ but X∉Cl(S). *S *being a solution for *I*, implies by Def. 4, that the aggregated deep coalescence cost, i.e. $\sum_{i=1}^{n}DC\left(T_{i},\;S\right)$ is minimized. Then, based on the existence of the cluster *X*, we edit *S *and form a new tree *R *using a tree edit operation which will be introduced in the next section. The properties of this new operation together with the properties of *X *(proved in the next section), provides the key ingredients to calculate the changes in deep coalescence costs. With some further arithmetics, this allows us to conclude that *R *in fact has a smaller aggregated deep coalescence cost, i.e. $\sum_{i=1}^{n}DC\left(T_{i},\;S\right)>\sum_{i=1}^{n}DC\left(T_{i},\;R\right)$, hence contradicting the assumption that *S *is a solution for *I*.

### Supporting lemmata

#### Shallowest regrouping operation

**Figure 3:**
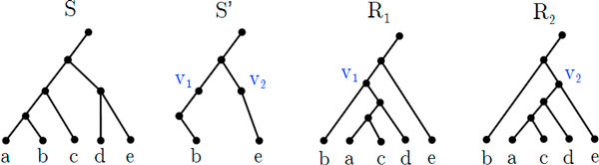
**Example of the shallowest regrouping operation**. Example of the shallowest regrouping operation of *S *by *X *where *X *= {*a, c, d*}. The intermediate tree $S^{\prime }=S(\bar{X)}$ shows its two shallowest degree-two nodes *v*_1_ and *v*_2_. *R*_1_ and *R*_2_ are the resulting trees of this operation. That is, $\hat{\Gamma }\left(S,X\right)=\left\{R1,R2\right\}$ where *R*_1_ = Γ(*S, X, v*_1_) and *R*_2_ = Γ(*S, X, v*_2_).

In this section we formally define the new tree edit operation that forms the key part of the theorem. We begin with some useful definitions related to the depth of nodes. An example of this operation is shown in Figure 3.

**Definition 8 **(Node depth). *The *depth *of a node v*∈*V*(*T*), *denoted dep_T _*(*v*)*, is *$pl\left(v,\text{ Ro}\left(T\right)\right)$.

**Definition 9 **(Shallowest nodes). *Let T be a tree and X *⊆ *V*(*T*), *the *shallowest *function, denoted shallowest_T_*(*X*), *is the set of nodes in X which have the minimum depth among all nodes in X*. *Formally, we define shallowest*_T _(*X*) ≜ *argmin_v∈X _*(*dep_T_*(*v*)).

Now we have the necessary mechanics to define the new tree edit operation. In what follows, we assume *S *to be a tree, ∅⊂X⊂Le(S), and $S\prime =\;S\left(\bar{X}\right)$.

**Definition 10 **(Regroup). *Let v *∈ *I*_2_(*S*'). *The regrouping operation of S by X on v, denoted *Γ(*S, X, v*), *is the tree obtained from S' by*

*1. (R1) Identify *Ro(*S*|*X*) *and v. In other words we adjoin the root of tree S|X onto the node v*.

*2. (R2) Suppress all nodes with degree two*.

**Definition 11 **(Shallowest regroup). *The *shallowest regrouping *operation of S by X, denoted *$\hat{\Gamma }\left(S,\;X\right),$*defines a set of trees by *$\hat{\Gamma }\left(S,\;X\right)≜\left\{\Gamma \left(S,\;X,\;v\right):v\in shallowest_{S\prime }\;\left(I_{2}\left(S\prime \right)\right)\right\}.$

As Figure 3 shows, the shallowest regrouping operation pulls apart *X *from *S *and regroups *X *back onto each of the shallowest nodes in *S*.

#### Counting the number of degree-two nodes

The regrouping operation includes the step of suppressing nodes with degree two. Since this step affects path lengths and ultimately deep coalescence costs, we are required to count carefully the number of degree-two nodes under various conditions. Here we assume that *T *is a tree and {*X, Y*} is a bipartition of Le(T). We begin with two observations that assert existence of degree-two nodes, and assert existence of leaf sets given a degree-two node.

**Observation 1**. *I*_2_(*T*(*X*) ≠ Ø *and*. *I*_2_(*T*(*Y*) ≠ Ø.

**Observation 2**. *If v *∈ *I*_2_(*T*(*X*)), *then *$Le\left(T_{v}\right)\cap X\neq \emptyset$ and. $Le\left(T_{v}\right)\cap Y\neq \emptyset$.

The next Lemma says that if the root of *T *is the parent of *lca*(*X*), then the number of degree-two nodes in *T*(*X*) is at least the depth of *v*, where *v *is a shallowest degree-two node of *T*(*Y*).

**Lemma 1**. *If *$Pa\left(lca\left(X\right)\right)=Ro\left(T\right)$*and v *∈ *shallowest *(*I*_2_(*T*(*Y*))), *then dep*(*v*) ≤ |*I*_2_(*T*(*X*))|.

**Figure 4:**
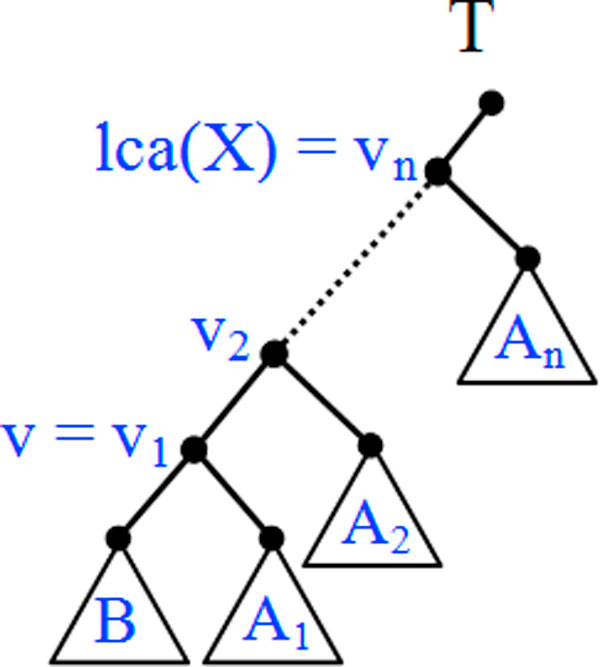
**Setup and variable assignments for the proof of Lemma 1**. Tree showing the variable assignments in the proof of Lemma 1. Dotted lines represent omitted parts of the tree, and triangles represent subtrees.

*Proof*. Assume the premise. Let *n *= *dep*(*v*), we observe that *n *≥ 1 because of the root edge. Figure 4 shows the setup and variable assignments for this proof. Let *v *= *v*_1_ < ... <*v_n_*, and let *B, A*_1_, ... , *A_n _*be the leaf sets of the indicated subtrees. We observe the following:

• *v_n _*= *lca*(*X*) because $Pa\left(lca\left(X\right)\right)\;=Ro\left(T\right)$.

• *B *⊆ *X *and *A*_1_ ∩ *Y *≠ ∅, because *v *is a degree-two node of *T*(*Y*).

• *A*_1_ ∩ *Y *≠ ∅ implies that *A*_2_,..., *A_n _*each contains at least an element of *Y*. For otherwise, each of *v*_2_, ...,*v_n _*becomes a degree-two node in *T*(*Y*), contradicting the assumption that *v *= *v*_1_ is the shallowest degree-two node in *T*(*Y*).

In order to obtain *T*(*X*), we must prune subtrees in *A*_1_ whose leaves are in *Y *(which could be the entire subtree *A*_1_). Thus there must be at least one degree-two node in *A*_1_ (or *v*_1_ if *A*_1_ is pruned). Similarly, for 1 <*i *≤ *n*, either *v_i _*has degree two or there exists a degree-two node in *A_i_*. Overall *T*(*X*) has at least *n *degree-two nodes, as required. □

#### Properties of the regrouping operation

We examine some properties of the regrouping operation in this section. In general, these properties show that the path lengths defined by LCA's do not increase under several different assumptions. This preservation of path lengths would later assist in the calculation of deep coalescence costs. Throughout this section, we assume *S *to be a tree, ∅⊂X⊂Le(S), and $S^{\prime }=S\left(\bar{X}\right).$ Further we let *R *= Γ(*S, X, v*) where *v *∈ *I*_2_(*S*').

**Lemma 2**. *If *A⊆B⊆Le(S)*and *$B\subseteq \;\bar{X}$* , then pl_S_*(*A, B*) = *pl_S' _*(*A, B*).

**Lemma 3**. *If *A⊆B⊆Le(S)*and *$B\subseteq \;\bar{X}$*, then pl_S_*(*A, B*) ≥ *pl_R_*(*A, B*).

**Lemma 4**. *If *A⊆B⊆Le(S)*and *B⊆X* , then pl_S_*(*A, B*) ≥ *pl_R_*(*A, B*).

**Lemma 5**. *If A⊆B⊆Le(S)*, $A\subseteq \;\bar{X}$* , and X *⊆ *B, then pl_S_*(*A, B*) ≥ *pl_R_*(*A, B*).

*Proof*. Let *S*" be the tree obtained from *S*' by identifying Ro(*S*|*X*) and *v*. In other words, *S*" is the tree after step (R1) of the regroup operation Γ(*S, X, v*). We will show that *pl_S _*≥ (*A, B*) ≥ *pl_S" _*(*A, B*) ≥ *pl_R_*(*A, B*). We begin with the first inequality. First, since $A\subseteq \;\bar{X}$ we know that *lca_S_*(*A*) = *lca_S'_*(*A*) = *lca_S" _*(*A*). Let *x *= *lca_S_*(*X*) and *b *= *lca_S_*(*B*), then the assumption of *X *⊆ *B *implies *x *≤*_S_ b*. Since *v *has degree two in *S*', we know that $Le\;\left(S_{v}\right)\cap X\neq \;\emptyset$ (Observation 2), and so *v *≤*_S_ x*. Now let *x*" = *lca_S" _*(*X*) and *b*" = *lca_S"_*(*B*). By (R1) we have that *x" *≤ *_S"_ v*, and so *x*" ≤ *_S" _x*, which implies *b*" ≤ *_S" _b*. Furthermore, *lca_S_*(*A*)= *lca_S" _*(*A*) is a descendant of both *b *and *b*" because *A *⊆ *B*, and hence *b*" ≤ *_S" _ b *implies that *pl_S_*(*A, B*) ≥ *pl_S" _*(*A, B*).

Next, by (R2) *R *is obtained from *S*" by suppressing some nodes, therefore a path in *S*" can only be made shorter in *R*, hence we have *pl_S"_*(*A, B*) ≥ *pl_R_*(*A, B*).

Finally, combining the above results we have *pl_S_*(*A, B*) ≥ *pl_R_*(*A, B*). □

### Main theorem

**Theorem 1**. *Deep coalescence consensus tree problem is Pareto on clusters*.

*Proof*. Assume not for a contradiction, then there exists an instance *I *= (*T*_1_,...,*T_n_*), a solution *S *for *I*, and a cluster X⊆Le(S) where $X\in \cap_{i=1}^{n}Cl\text{(}T_{i}\text{)}$ but $X\notin Cl\left(S\right)$. Since $X\notin Cl\left(S\right)$, *X *must be non-trivial, therefore $\hat{\Gamma }\left(S,\;X\right)$ does not contain *S *and is not empty. Let $R\in \hat{\Gamma }\left(S,\;X\right)$. We will show that (∀ 1 ≤ *i *≤ *n*) (*DC*(*T_i_, S*) >*DC*(*T_i_, R*)), which implies $\sum_{i=1}^{n}DC\left(T_{i},\;S\right)\;>\;\sum_{i=1}^{n}DC\left(T_{i},\;R\right),$ contradicting the assumption that *S *is a solution for *I*.

Let *T *= *T_i _*where 1 ≤ *i *≤ *n*, we will show that *DC*(*T, S*) >*DC*(*T, R*). This requires that *DC*(*T, S*) - *DC*(*T, R*) > 0, in other words

(1) $$\underset{\begin{array}{c} \left\{u,v\right\}\in E\left(T\right)\\ u<v \end{array}}{\sum }\left(pl_{S}\left(Le\left(T_{u}\right),\;Le\left(T_{v}\right)\right)-pl_{R}\left(Le\left(T_{u}\right),\;Le\left(T_{v}\right)\right)\right)>0$$

**Figure 5:**
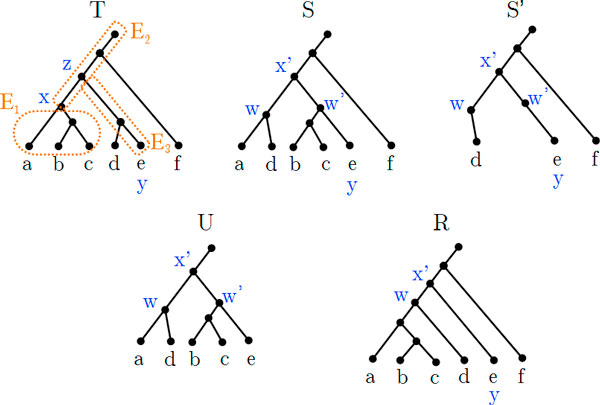
**Running example for the proof of Theorem 1**. A running example for the proof of Theorem 1 where *T *is a tree in the instance tuple *I, S *is an assumed solution for $I,X=\left\{a,b,c\right\},\;\;S^{\prime }=S\left(\bar{X}\right),\;\;U=Sx^{\prime }$, and *R *= Γ(*S, X, w*). Highlighted regions in *T *are the edge partitions *E*_1_, *E*_2_, and *E*_3_. The rest of edges form the partition *E*_4_. By counting the costs for each partition we have Σ_1_ =6 - 4 = 2, Σ_2_ =1 - 3 = −2, Σ_3_ = 2 − 1 = 1, and Σ_4_ =3 − 3 = 0. Overall we have *DC*(*T, S*) − *DC*(*T, R*) = 1.

Since (1) sums over all edges in *T*, for convenience we partition the edges of *T *and compute the differences in path lengths for each partition individually. Figure 5 depicts a running example for *T, S*, and *R *where *X *= {*a, b, c*}.

We identify some specific nodes in order to partition the edges of *T*. Let $S^{\prime }=S\left(\bar{X}\right),\;w\in I_{2}\left(S^{\prime }\right)$ where *R *= Γ(*S, X, w*). Since $X\notin Cl\left(S\right)$, *S*' contains at least two nodes with degree two. Let *w*'∈ *I*_2_(*S*') such that *w*' ≠ *w*, then *S_w' _*contains some leaf *y *∉ *X *(Observation 2).

Let *x *= *lca_T _*(*X*) and *z *= *lca_T _*(*X *∪ {*y*}), we partition the edges of *T *into {*E*_1_, *E*_2_, *E*_3_, *E*_4_} as follows.

1. *E*_1_ ≜ {{*u, v*} ∈ *E*(*T*) : *u *<*v *≤ *x*} = A ll edges under *x*

2. *E*_2 _≜ {{*u, v*} ∈ *E*(*T*) : *x *≤ *u *<*v*} = Edges forming the path from *x *to Ro(T)

3. *E*_3_ ≜ {{*u,v*} ∈ *E*(*T*) : *y *≤ *u *<*v *≤ *z*} = Edges forming the path from *y *to *z*

4. *E*_4_ ≜ *E*(*T*) \ (*E*_1 _∪ *E*_2_ ∪ *E*_3_)

We consider (1) for each of the partition separately. For clarity, we define the aggregated cost difference Σ*_i _*for partition *E_i _*as follows.

$$\Sigma_{i}≜\underset{\begin{array}{c} \left\{u,v\right\}\in E_{i}\\ u<v \end{array}}{\sum }\left(pl_{S}\left(Le\left(T_{u}\right),\;Le\left(T_{v}\right)\right)-pl_{R}\left(Le\left(T_{u}\right),Le\left(T_{v}\right)\right)\right)$$

Hence (1) becomes

(2) $$\Sigma_{\text{1}}+\Sigma_{\text{2}}+\Sigma_{\text{3}}+\Sigma_{\text{4}}>0$$

Let *x*' = *lca_S_*(*X*) and *p *= *pl_S _*(*w, x*') + 1. For each *i *∈ {1, 2, 3, 4}, we claim and prove the bound of Σ*_i _*as follows.

**Claim 1**. Σ_1_ ≥ *p*

*Proof*. First we observe that the difference for each path length in this partition is ≥ 0 (Lemma 4), so we have Σ_1_ ≥ 0. Since *x*' = *lca_S _*(*X*), we only need to consider the subtree *S_x' _*in computing the path lengths in this partition. Define *U *= *S_x'_*. In particular, the number of degree two nodes in *U*(*X*) gives us a lower bound on the total decreases of path lengths, because these nodes are removed to obtain *U*|*X *which is a subtree of *R*. That is, Σ_1_ ≥ |*I*_2_(*U*(*X*))|. Lemma 1 applies to *U *with bipartition {X,Le(U)\X} and the node *w*, so we have |*I*_2_(*U*(*X*))| ≥ *dep_U _*(*w*). The depth *dep_U _*(*w*) is with respect to *U*, and we relate it to a path length in *S *by taking away the root edge, that is $dep_{U}\text{(}w\text{)}\;-\text{1 = }pl_{S}\text{(}w\text{, }x\prime \text{)}$. Finally, using the definition of *p *we obtain Σ_1_ ≥ |*I*_2_(*U*(*X*))| ≥ *dep_U _*(*w*)= *pl_S _*(*w, x*') +1= *p*.

**Claim 2**. Σ_2_ = −*p*

*Proof*.

$$\begin{array}{llll} \Sigma_{2} & =pl_{S}\left(X,Le\left(T\right)\right)-pl_{R}\left(X,Le\left(T\right)\right)\; & & \;\\ & =\left[pl_{S}\left(x\prime ,Ro\left(T\right)\right)-1\right]-\left[1+pl_{R}\left(w,x\prime \right)+pl_{R}\left(x\prime ,Ro\left(T\right)\right)-1\right]\; & & \;\\ & =pl_{S}\left(x\prime ,Ro\left(T\right)\right)-\left[1+pl_{R}\left(w,x\prime \right)+pl_{R}\left(x\prime ,Ro\left(T\right)\right)\right]\; & & \;\\ & =pl_{S}\left(x\prime ,Ro\left(T\right)\right)-\left[1+pl_{S}\left(w,x\prime \right)+pl_{S}\left(x\prime ,Ro\left(T\right)\right)\right]\; & & \;\\ & =-\text{[}pl_{S}\text{(}w\text{, }x\prime \text{) + 1]}\; & & \;\\ & =-p\; & & \;\\ & \; & \end{array}$$

The fourth equality holds because *w *is the shallowest degree-two node in *S*', so that no edges along the path from *w *to *x*' are contracted in *R*, hence *pl_R_*(*w, x*') = *pl_S_*(*w, x*').

**Claim 3**. Σ_3_ ≥ 1

*Proof*. Let {*a, b*} ∈ *E*_3_ where *a*<*_T_ b*, $A=Le\left(T_{a}\right)$, and $B=Le\left(T_{b}\right)$. We know that $A\subseteq \;\bar{X}$ because otherwise this edge should be in *E*_1_ or *E*_2_. We consider two cases for *B*.

1. If $B\subseteq \;\bar{X}$, then Lemma 3 applies on *S, R, A, B*, so *pl_S_*(*A, B*) − *pl_R_*(*A, B*) ≥ 0.

2. If *X *⊆ *B*, then Lemma 5 applies on *S, R, A, B*, so *pl_S_*(*A, B*) − *pl_R_*(*A, B*) ≥ 0.

In any case, we have *pl_S_*(*A, B*) − *pl_R_*(*A, B*) ≥ 0 for each edge {*a, b*} ∈ *E*_3_. This implies that Σ_3_ ≥ 0. Further, since *w*' ∈ *I*_2_(*S*') and *w*' ≠ *w, w*' does not exist in *R*. We also know that $y{<}_{S}w\prime {<}_{S}lca_{S}\left(X\cup \left\{y\right\}\right)$ by the definitions of *w*' and *y*. Therefore there exists an edge {*a, b*} ∈ *E*_3_ such that *pl_S_*(*A, B*) − *pl_R_*(*A, B*) ≥ 1. Hence we have Σ_3_ ≥ 1.

**Claim 4**. Σ_4_ ≥ 0

*Proof*. Let {*a, b*} ∈ *E*_4_ where *a *<*_T_ b*, $A=Le\left(T_{a}\right)$, and $B=Le\left(T_{b}\right)$. The proof follows from the same argument as in Claim 3 where we have *pl_S _*(*A, B*) − *pl_R_*(*A, B*) ≥ 0 for each edge {*a, b*} ∈ *E*_4_, hence Σ_4_ ≥ 0.

Finally, we have Σ_1_ +Σ_2_ +Σ_3_ +Σ_4_ ≥ *p *+ (-*p*) + 1+ 0 =1 > 0. Hence (2) is satisfied, and so is (1). In sum, we have constructed a tree *R *and showed that $\sum_{i=1}^{n}DC\left(T_{i},\;S\right)\;>\;\sum_{i=1}^{n}DC\left(T_{i},\;R\right),$ which contradicts with the assumption that *S *is a solution for *I*, in other words the assumption that *S *has the minimum aggregated cost with respect to the deep coalescence cost function. □ 

### Algorithm for improving a candidate solution

Algorithm 1 takes a consensus tree problem instance and a candidate solution as inputs. If the candidate solution does not display the consensus clusters, it is transformed into one that includes all of the consensus clusters and has a smaller (more optimal) deep coalescence cost.

**Algorithm 1 **Deep coalescence consensus clusters builder

1: **procedure **DCConsensusClustersBuilder (*I, T*)

Input: A consensus tree problem instance *I *= (*T*_1_,...,*T_n_*), a candidate solution *T *for *I*

Output: *T*, or an improved solution *R *that contains all consensus clusters of *I*

2:    *R *← *T*

3:    *C *← Set of all consensus clusters of *I*

4:    **for all **cluster *X *∈ *C ***do**

5:       **if ***R *does not contain *X ***then**

6:          *v *← *A *node in *shallowest *$\left(I_{\text{2}}\left(R\left(\bar{X}\right)\right)\right)$ (shallowest degree-two node of $R\left(\bar{X}\right)$)

7:          *R *← Γ(*R, X, v*) (regrouping operation of *R *by *X *on *v*)

8:       **end if**

9:       **end for**

10:    **return ***R*

11: **end procedure**

The correctness of Algorithm 1 follows from the proof of Theorem 1. We now analyze its time complexity. Let *m *be the number of taxa present in the input trees. Line 3 takes *O*(*nm*) time. Line 5, 6, and 7 each takes *O*(*m*) time, and there are *O*(*m*) iterations. Overall Algorithm 1 takes *O*(*nm *+ *m*^2^) time.

### General method for improving a search algorithm

**Figure 6:**
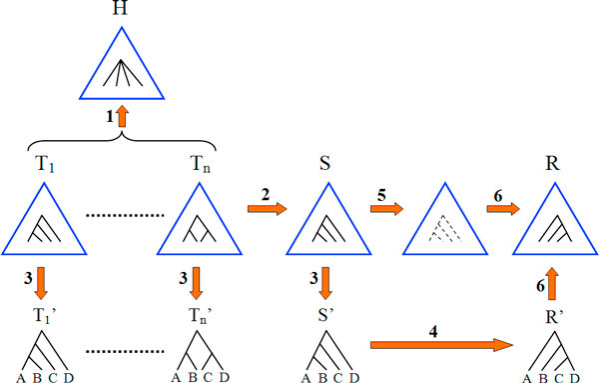
**Running example for the definitions and proof of Theorem 2**. A running example for the definitions and proof of Theorem 2. Arrows are marked by numbers 1 to 6, demonstrating the steps of the proof. Each step is explained below: (1) Given an instance *I *= (*T*_1_,...,*T_n_*), let *H *be the strict consensus tree of *I*. An internal node of *H *and its four children are shown. (2) Let *S *be a solution for *I*, having the optimal deep coalescence cost. (3) Cut trees via *H *and *h*, obtaining $Cu{\text{t}}_{H,h}\left(I\right)$ and $Cu{\text{t}}_{H,h}\left(S\right)=S^{\prime }.$ Let *A, B, C, D *be the leaf sets of each subtree. (4) We assume by contradiction that *S*' is not a solution for $Cu{\text{t}}_{H,h}\left(I\right)$, and so we let *R*' be a solution for $Cu{\text{t}}_{H,h}\left(I\right)\text{.}$ (5 and 6) Modify *S *to obtain *R*, by replacing *S*' with *R*'.

In this section we extend the result of Theorem 1 and show that the deep coalescence consensus tree problem exhibits optimal substructures based on the *strict consensus tree *of the problem instance. This leads to another simple and general method that improves an existing search algorithm. Figure 6 depicts a running example for this section. We now begin with some useful definitions.

**Definition 12 **(Strict consensus tree [18]). *Given a tuple of n trees I *= (*T*_1_,...,*T_n_*), *the *strict consensus tree of *I, denoted StrictCon*(*I*), *is the unique tree that contains those clusters common to all the input trees. Formally, StrictCon*(*I*) *is a *(*possibly non-binary*) *tree S such that *$Cl\left(S\right)={⋂}_{i=1}^{n}Cl\left(T_{i}\right)$.

**Definition 13 **(Cut on trees). *Let H and T be two trees over the same leaf set, such that H is a non-binary tree and T is a binary tree that refines H. Given an internal node h in H, a *cut *on T via H and h, denoted *$Cu{\text{t}}_{H,h}\left(T\right)$*, is the minimal connected subtree of T that contains *$\left\{M_{H⊳T}\left(c\right):c\in {Ch}_{H}\left(h\right)\right\}$, *and we rename each leaf x by *$Le\left(T_{x}\right)$.

*We further extend this to a tuple of trees I *= (*T*_1_,...,*T_n_*) by ${Cut}_{H,h}\left(I\right)≜\left({Cut}_{H,h}\left(T_{1}\right),\ldots ,{Cut}_{H,h}\left(T_{n}\right)\right)$.

**Theorem 2**. *Let I *=(*T*_1_,...,*T_n_*) *be an instance of the deep coalescence consensus tree problem, and let S be a solution for I (having the optimal deep coalescence cost). Further suppose H is the strict consensus tree of I, and h is an internal node in H. Then *$Cu{\text{t}}_{H,h}\left(S\right)$*is a solution for the instance *$Cu{\text{t}}_{H,h}\text{(}I\text{)}$*of the deep coalescence consensus tree problem*.

*Proof*. Let $Cu{\text{t}}_{H,h}\left(S\right)=S\text{'}$ and $Cu{\text{t}}_{H,h}\left(I\right)=\left(Cu{\text{t}}_{H,h}\left(T_{1}\right),\ldots ,Cu{\text{t}}_{H,h}\left(T_{n}\right)\right)=\left(T_{1}^{\prime },\ldots ,T_{n}^{\prime }\right)$. First we observe that *S *must be a refinement of *H *by Theorem 1, therefore *S' *is defined. We continue to prove by contradiction, assuming the premise holds but *S' *is not a solution for the instance ${Cut}_{H,h}\left(I\right)$. So let *R' *be a solution for the instance $Cu{\text{t}}_{H,h}\text{(}I\text{)}$, this implies that $\sum_{i=1}^{n}DC\left({T^{\prime }}_{i},\;S^{\prime }\right)>\sum_{i=1}^{n}DC\left({T^{\prime }}_{i},\;R^{\prime }\right)$. We now modify *S *by replacing *S' *with *R' *as follows:

1. Remove all edges of *S*', and remove all nodes of *S*' excepts the root and the leaves.

2. Identify $Ro\left(S^{\prime }\right)$ with. $Ro\left(R^{\prime }\right)$

3. For each leaf *v *of *S'*, identify *v *with a leaf *x *of *R' *where $x=Le\left(S_{v}\right)$.

Let the resulting tree be *R*. We will show that *R *has a lower deep coalescence cost, contradicting the assumption that *S *is a solution for *I*.

Let *T *= *T_i _*where 1 ≤ *i *≤ *n*, it suffices to show that *DC*(*T,S*) >*DC*(*T, R*), in other words

(3) $$\underset{\begin{array}{c} \left\{u,v\right\}\in E\left(T\right)\\ u<v \end{array}}{\sum }\left(pl_{S}\left(M_{T⊳S}\left(u\right),\;M_{T⊳S}\left(v\right)\right)-pl_{R}\left(M_{T⊳R}\left(u\right),\;M_{T⊳R}\left(v\right)\right)\right)>0$$

For convenience, let ${Ch}_{H}\left(h\right)=\left\{c_{1},\ldots ,c_{m}\right\},h^{\prime }=M_{H⊳T}\left(h\right)$, and $c_{j}^{\prime }=M_{H⊳T}\left(c_{j}\right)$where 1 ≤ *j *≤ *m*.

Similar to the proof of Theorem 1, we partition the edges of *T *into {*E_under_ , E_out_, E_in_*} as follows.

1. $E_{under}≜\left\{\left\{u,\;v\right\}\in E\left(T\right):u<v\text{ and }\;\left(\exists j\right)\left(v\leq c_{j}^{\prime }\right)\right\}$

2. $E_{out}≜\left\{\left\{u,\;v\right\}\in E\left(T\right):u<v\text{ and }\;v≰h^{\prime }\right\}$

3. $E_{in}≜E\left(T\right)\backslash \left(E_{under}\cup E_{out}\right)$

Recall that the modification of *S *into *R *only involves the subtree *S*', therefore $M_{T⊳S}\left(v\right)$ is unchanged for every *v *occurs in *E_under _*and *E_out_*. Hence it suffices to evaluate (3) on *E_in _*only. However we have already assumed that $\sum_{i=1}^{n}DC\left({T^{\prime }}_{i},\;S^{\prime }\right)>\sum_{i=1}^{n}DC\left(T_{i}^{\prime },\;R^{\prime }\right)$, therefore (3) holds. Overall we have that *R *has a lower deep coalescence cost, contradicting the assumption that *S *is a solution for *I*. □

Theorem 2 implies that every internal node of the strict consensus tree defines an independent subproblem, and solutions of these subproblems can be combined to give a solution to the original deep coalescence consensus tree problem. This leads to the following general divide and conquer method that improves an existing search algorithm.

**Method 1 **Deep coalescence consensus tree method

1: **procedure **DCConsensusTreeMethod(*I*)

Input: A DC consensus tree problem instance *I *=(*T*_1_,...,*T_n_*), an external program DC-SOLVER.

Output: A candidate solution *T *for *I*

2:    *H *← *StrictCon*(*I*)

3:    **for all **internal node *h *of *H ***do**

4:       *I_h _*← $Cu{\text{t}}_{H,h}\text{(}I\text{)}$

5:       *S_h _*← DC-SOLVER(*I_h_*)

6:       Refine the children of *h *on *H *by the tree *S_h_*

7:    **end for**

8:    **return ***H*

9:  **end procedure**

## Results

We used simulation experiments to (i) test if the solutions obtained from efficient heuristics presented in [13] display the Pareto property, and (ii) compare the performance of our new divide and conquer approach based on the Pareto property to the generic heuristic in [13].

### Experiment results 1

First to examine if subtree pruning and regrafting (SPR) heuristic solutions from [13] display the Pareto property, we generated a series of four 14-taxon trees that share few clusters. To do this, we first generated random 11-taxon trees. Next, we generated random 4-taxon trees containing the species 11-14. We then replaced the one of the leaves in the 11-taxon tree with the random 4-taxon tree. This procedure produces gene trees that share at least a single 4-taxon cluster in common. Although this simulation does not reflect a biological process, it represents extreme cases of error or incongruence among gene trees. In three cases with the 14-taxon gene trees, we found that the SPR heuristic did not return a result that contained the consensus cluster. In these cases, our proof demonstrates that there exists a better solution that also contained the consensus cluster. However, the failure of the SPR heuristic in these cases appears to depend on the starting tree; these data sets did not fail with all starting trees. Thus, the shortcomings of the SPR heuristic may be ameliorated by performing multiple runs from different starting trees.

### Experiment results 2

**Figure 7:**
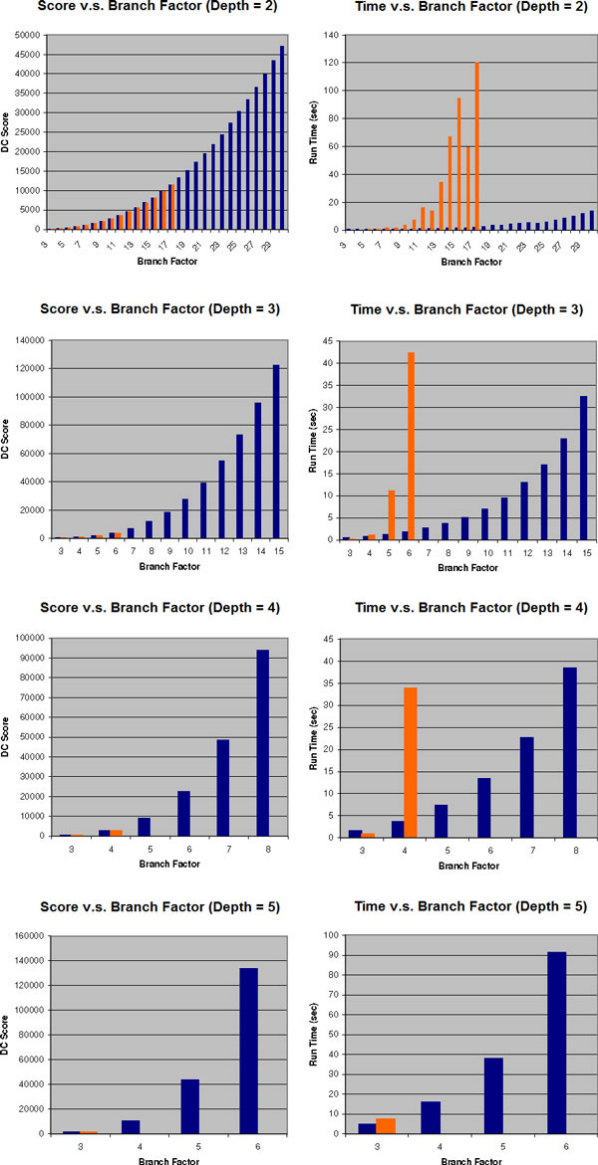
**Deep coalescence score and runtime results for Experiment 2**. Legend: blue represents Method 1 (divide and conquer) and orange represents standalone SPR heuristic.

We next evaluated the efficacy and scalability of Method 1 and compared it to the standalone SPR heuristic. We generate sets of gene trees, each with different consensus tree structures (depths and branch factors) as follows. The *depth *of a tree is the maximum number of edges from the root to a leaf, and the *branch factor *of a tree is the maximum degree of the nodes. For each depth *d *and a branch factor *b*, we first generate a complete *b*-ary tree of depth *d*, denoted *C_d,b_*. This tree represents the consensus tree. We used depths of 2-5, and branch factors of 3-30. For each *C_d,b_*, we then generated 10 sets of 20 random gene trees, such that each gene tree is a binary refinement of *C_d,b_*. Each set of input trees was given as input to Method 1, using [13] as the external deep coalescence solver. For comparison, we ran the same data sets using [13] as the standalone deep coalescence solver. We calculated the deep coalescence score for each output species tree, and we report the average score of 10 profiles as the score for each *C_d,b_*. We also measured and recorded the average runtime of each run. We terminate the execution of the standalone solver if the runtime exceeds two minutes, and in this case the results are not shown. In general, Figure 7 shows that the scores of the trees were very similar from Method 1 and the standalone SPR heuristic. Thus, Method 1 does not appear to improve the quality of the deep coalescence species trees. However, Method 1 shows extreme improvements in the runtime, especially as the branch factors increase.

### Experiment results 3

Finally, we examined the performance of Method 1 and compare it to the standalone SPR heuristic using more biologically plausible coalescence simulations. We followed the general structure the coalescence simulation protocol described by Maddison and Knowles [9]. First, we generated 40 256-taxon species trees based on a Yule pure birth process using the r8s software package [19]. To transform the branch lengths from the Yule simulation to represent generations, we multiplied them all by 1,000,000. Next, we simulated coalescence within each species tree (assuming no migration or hybridization) using Mesquite [20]. All simulations produced a single gene copy from each species. For each species tree, we simulated 20 gene trees assuming a constant population size. The population size effects the number of deep coalescence events, with larger populations leading to more incomplete lineage sorting and consequently less agreement among the gene trees. Thus, to incorporate different levels of incomplete lineage sorting, for 20 of the species trees, we used a constant population size of 10,000, and for 20 we used a constant population size of 100,000. Thus, in total, we produced 40 sets of 20 gene trees, with each set simulated from a different 256-taxon species tree.

For each data set, we performed a phylogenetic analyses using Method 1 and also using only the SPR heuristic from Bansal et al. [13]. In contrast to the simulations in Experiment 1, the standalone SPR heuristic of Bansal et al. [13] always returned species trees with all consensus clusters. Of course, all solutions from Method 1 must display the Pareto property. The deep coalescence reconciliation score for the best trees were similar with both algorithms. When the population size was 10,000, the average coalescence cost was 279, and all the gene trees shared an average of 29.4 clusters. In 19 out of the 20 of these simulations, both approaches produced the same results, while in one case, Method 1 found a species tree with a one fewer implied deep coalescence event. When the population size was 100,000, the average coalescence cost was 2142, and the all gene trees shared an average of 19.1 clusters. Although the reconciliation cost never differed by more than 15, Method 1 had a better score in 6 replicates, and the standalone SPR had a better score in 11 replicates. All analyses finished within 30 seconds in a laptop PC, but Method 1 was always faster than SPR alone.

## Discussion

In addition to offering a biologically informed optimality criterion to resolve incongruence among gene trees, we prove that the deep coalescence problem also is guaranteed to retain the phylogenetic clusters for which all gene trees agree. Since the deep coalescence problem is NP-hard [10], most meaningful instances will require heuristics to estimate a solution. We demonstrate that the Pareto property can be leveraged to vastly improve upon the running time of heuristics. Method 1 represents a new general approach to phylogenetic algorithms. In most cases, heuristics to estimate solutions for phylogenetic inference problems are based on a few generic search strategies such as the local search heuristics based on nearest neighbor interchange (NNI), SPR, or tree bisection and reconnection (TBR) branch swapping. Although these search strategies often appear to perform well, they are not connected to any specific phylogenetic problems or optimality criteria. Ideally, however, efficient and effective heuristics should be tailored to the properties of the phylogenetic problem. In the case of the deep coalescence consensus tree problem, the Pareto property provides an informative guiding constraint for the tree search. Specifically, when considering possible solutions, we need only consider solutions that contain all clusters from the input gene trees, or, in other words, that refine the strict consensus of the input gene trees.

Still, our simulation experiments suggest that, in many cases, the SPR local search heuristic described by Bansal et al. [13] performs well. While we identified cases in which the estimate from the SPR heuristic did not contain the Pareto clusters, in most cases SPR alone found trees as good, or even slightly better, than Method 1. We note that the size of the simulated coalescence data set, 256 taxa, exceeds the size of the largest published analysis of the deep coalescence consensus tree problem and is far beyond the largest instances (8 taxa) from which exact solutions have been calculated [11], and the SPR found good solutions within 30 seconds. Still, running time for the SPR heuristic does not always scale well, and the results of Experiment 2 suggest that it might not be tractable for extremely large data sets. In these cases, in practice Method 1 may vastly improve upon the running time, while guaranteeing a solution with the Pareto property.

Further, Theorem 2 shows that the deep coalescence consensus tree problem exhibits independent optimal substructures. This implies that, once we compute the strict consensus tree of the problem instance, the rest of Method 1 can be directly parallelized, regardless of which external deep coalescence solver is used. In the case where the external solver guarantees exact solutions, our method would also give exact solutions, but can potentially solve instances with a much larger taxa size compared to running the external solver alone.

Although the Pareto property for the deep coalescence consensus tree problem is desirable, and the divide and conquer method is promising for large-scale analyses, there are limitations to their use. First, the Pareto property and Method 1 are limited to the consensus case, or, instances in which all of the input gene trees contain sequences from all of the species. Also, the Pareto property is only useful when all input trees share some clusters in common. If there are no consensus clusters among the input trees, then Method 1 conveys no run-time benefits. While this may seem like an extreme case, it is possible with high levels of incomplete lineage sorting, or, perhaps more likely, much error in the gene tree estimates. Also, as we add more and more gene trees, we would expect more instances of conflict among the gene trees, potentially converging towards the elimination of consensus clusters. Than and Rosenberg [21] recently proved the existence of cases in which the deep coalescence problem is inconsistent, or converges on the wrong species tree estimate with increasing gene tree data. Although inconsistency is concerning, the Pareto property provides some reassurance. Even in a worse case scenario in which the deep coalescence problem is misled, the optimal solutions will still contain all of the agreed upon clades from the gene trees. Perhaps the greatest advantage of the deep coalescence problem, especially compared to likelihood and Bayesian approaches that infer species trees based on coalescence models (e.g., [22-24]), is its computational speed and the feasibility of estimating a species tree from large-scale genomic data sets representing hundreds or even thousands of taxa [13]. Not only can our method improve the performance of any existing heuristic, the Pareto property describes a limited subset of possible species trees that must contain the optimal solution.

## Conclusions

We prove that the deep coalescence consensus tree problem satisfies the Pareto property for clusters and describe an efficient algorithm that, given a candidate solution that does not display the consensus clusters, transforms the solution so that it includes all the consensus clusters and has a lower deep coalescence cost. We extend the result and prove that the problem exhibits optimal substructures based on the strict consensus tree of the input gene trees. Based on this property, we suggest a new, parallelizable tree search method, in which we refine the strict consensus of the input gene trees. In contrast to previously proposed heuristics, this method guarantees that the proposed solution will contain the Pareto clusters. Also, as our experiments demonstrate, this method can greatly improve the speed of deep coalescence tree heuristics, potentially enabling efficient and effective estimates from input with thousands of taxa.

## List of abbreviations used

LCA: least common ancestor; SPR: subtree pruning and regrafting; NNI: nearest neighbor interchange; TBR: tree bisection and reconnection

## Competing interests

The authors declare that they have no competing interests.

## Authors' contributions

HTL and OE were responsible for theory development and algorithm design. HTL implemented the programs. HTL and JGB designed and conducted simulation experiments, and JGB led the analysis of the results. All authors contributed to the writing of this manuscript, and have read and approved the final manuscript.

## Supplementary Material

Additional file 1 **Omitted proofs in the main manuscript**. Click here for file

## References

[B1] Rokas A Williams BL King N Carroll SB Genome-scale approaches to resolving incongruence in molecular phylogenies Nature 2003 425 6960 798 804 10.1038/nature02053 14574403

[B2] Pollard DA Iyer VN Moses AM Eisen MB Widespread Discordance of Gene Trees with Species Tree in Drosophila: Evidence for Incomplete Lineage Sorting PLoS Genet 2006 2 10 e173. 17132051 10.1371/journal.pgen.0020173 PMC1626107

[B3] Goodman M Czelusniak J Moore GW Romero-Herrera AE Matsuda G Fitting the Gene Lineage into its Species Lineage, a Parsimony Strategy Illustrated by Cladograms Constructed from Globin Sequences Systematic Zoology 1979 28 2 132 163 10.2307/2412519

[B4] Maddison WP Gene Trees in Species Trees Systematic Biology 1997 46 3 523 536 10.1093/sysbio/46.3.523

[B5] Nichols R Gene trees and species trees are not the same Trends in Ecology & Evolution 2001 16 7 358 364 10.1016/S0169-5347(01)02203-0 11403868

[B6] Edwards SV Is a new and general theory of molecular systematics emerging? Evolution; International Journal of Organic Evolution 2009 63 1 19 10.1111/j.1558-5646.2008.00549.x 19146594

[B7] Knowles LL Estimating Species Trees: Methods of Phylogenetic Analysis When There Is Incongruence across Genes Systematic Biology 2009 58 5 463 467 10.1093/sysbio/syp061 20525600

[B8] Yu Y Warnow T Nakhleh L Algorithms for MDC-based multi-locus phylogeny inference Proceedings of the 15th Annual international conference on Research in computational molecular biology 2011 RECOMB, Berlin, Heidelberg: Springer-Verlag 531 545

[B9] Maddison WP Knowles LL Inferring Phylogeny Despite Incomplete Lineage Sorting Systematic Biology 2006 55 21 30 10.1080/10635150500354928 16507521

[B10] Zhang L From gene trees to species trees II: Species tree inference in the deep coalescence model IEEE/ACM Trans Comput Biol Bioinformatics 2011 8 6 1685 1691 10.1109/TCBB.2011.83 21576759

[B11] Than C Nakhleh L Species Tree Inference by Minimizing Deep Coalescences PLoS Computational Biology 2009 5 9 e1000501 10.1371/journal.pcbi.1000501 19749978 PMC2729383

[B12] Than C Nakhleh L Estimating species trees: Practical and Theoretical Aspects Wiley-VCH, Chichester 2010 chap. Inference of parsimonious species tree phylogenies from multi-locus data by minimizing deep coalescences 79 98

[B13] Bansal M Burleigh JG Eulenstein O Efficient genome-scale phylogenetic analysis under the duplication-loss and deep coalescence cost models BMC Bioinformatics 2010 11 Suppl 1 S42 10.1186/1471-2105-11-S1-S42 20122216 PMC3009515

[B14] Bininda-Emonds ORP Phylogenetic supertrees: combining information to reveal the Tree of Life 2004 Springer

[B15] Bryant D A classification of consensus methods for phylogenies BioConsensus, DIMACS. AMS 2003 163 184

[B16] Wilkinson M Cotton JA Lapointe F Pisani D Properties of Supertree Methods in the Consensus Setting Systematic Biology 2007 56 2 330 337 10.1080/10635150701245370 17464887

[B17] Wilkinson M Thorley J Pisani D Lapointe FJ McInerney J Phylogenetic Supertrees: Combining Information to Reveal the Tree of Life Springer, Dordrecht, the Netherlands 2004 chap. Some desiderata for liberal supertrees 227 246

[B18] McMorris FR Meronk DB Neumann DA A view of some consensus methods for trees Numerical Taxonomy 1983 122 125

[B19] Sanderson MJ r8s: inferring absolute rates of molecular evolution and divergence times in the absence of a molecular clock Bioinformatics (Oxford, England) 2003 19 2 301 302 10.1093/bioinformatics/19.2.301 12538260

[B20] Maddison WP Maddison D Mesquite: a modular system for evolutionary analysis 2001 http://mesquiteproject.org

[B21] Than CV Rosenberg NA Consistency properties of species tree inference by minimizing deep coalescences Journal of Computational Biology 2011 18 1 15 10.1089/cmb.2010.0102 21210728

[B22] Liu L BEST: Bayesian estimation of species trees under the coalescent model Bioinformatics 2008 24 21 2542 2543 10.1093/bioinformatics/btn484 18799483

[B23] Kubatko LS Carstens BC Knowles LL STEM: species tree estimation using maximum likelihood for gene trees under coalescence Bioinformatics 2009 25 7 971 973 10.1093/bioinformatics/btp079 19211573

[B24] Heled J Drummond AJ Bayesian Inference of Species Trees from Multilocus Data Molecular Biology and Evolution 2010 27 3 570 580 10.1093/molbev/msp274 19906793 PMC2822290

