# Supplementary material for: Consensus properties for the deep coalescence problem and their application for scalable tree search
Source: BMC Bioinformatics. 2012 Jun 25;13(Suppl 10):S12. doi: 10.1186/1471-2105-13-S10-S12 (PMC3382448; doi:10.1186/1471-2105-13-S10-S12)
Supplement: Additional file 1 — Omitted proofs in the main manuscript. [file 1471-2105-13-S10-S12-S1.pdf]

Omitted proofs in the main manuscript

Harris T. Lin[1], J. Gordon Burleigh[2] and Oliver Eulenstein*[1]

[1]Department of Computer Science, Iowa State University, Ames, IA, USA
[2]National Evolutionary Synthesis Center, Durham, NC, USA; University of Florida, Gainesville, FL, USA

Email: Harris T. Lin - htlin@iastate.edu; J. Gordon Burleigh - gburleigh@ufl.edu; Oliver Eulenstein*- oeulenst@iastate.edu;

*Corresponding author

A Relationship between deep coalescence and extra lineage

We show the differences in our definition of *deep coalescence* given in Def. 3 and its original definition termed *extra lineage* given in [1].

Definition 1. *The* Boolean value *of a statement ϕ, denoted as $[\![\phi]\!]$, is 1 if ϕ is true, 0 otherwise.*

Definition 2 (Edge Coverage)**.** *Let $\{u', v'\} \in E(S)$ and $u' < v'$, the* edge coverage *of $\{u', v'\}$ from T, denoted $C_{T \triangleright S}(u', v')$, is defined by*

$$C_{T \triangleright S}(u', v') \triangleq \sum_{\substack{\{u,v\} \in E(T) \\ u<v}} [\![M_{T \triangleright S}(u) \leq u' < v' \leq M_{T \triangleright S}(v)]\!]$$

Definition 3 (Extra Lineage [1])**.** *The* extra lineage cost *from T to S, denoted $EL(T, S)$, is*

$$EL(T, S) \triangleq \sum_{\substack{\{u',v'\} \in E(S) \\ u'<v'<\mathrm{Ro}(S)}} \Big(C_{T \triangleright S}(u', v') - 1 \Big)$$

Proposition 1. $EL(T, S) = DC(T, S) - |E(S)| + 1$

Proof.

$$\begin{aligned}
EL(T,S) &= \sum_{\substack{\{u',v'\}\in E(S)\\ u'<v'<\mathtt{Ro}(S)}} \Big(C_{T\triangleright S}(u',v')-1\Big)\\
&= \Big(\sum_{\substack{\{u',v'\}\in E(S)\\ u'<v'<\mathtt{Ro}(S)}} C_{T\triangleright S}(u',v')\Big) - \Big(\sum_{\substack{\{u',v'\}\in E(S)\\ u'<v'<\mathtt{Ro}(S)}} 1\Big)\\
&= \Big(\sum_{\substack{\{u',v'\}\in E(S)\\ u'<v'<\mathtt{Ro}(S)}} \sum_{\substack{\{u,v\}\in E(T)\\ u<v}} [\![M_{T\triangleright S}(u)\le u'<v'\le M_{T\triangleright S}(v)]\!]\Big) - |E(S)|+1\\
&= \Big(\sum_{\substack{\{u,v\}\in E(T)\\ u<v}} \sum_{\substack{\{u',v'\}\in E(S)\\ u'<v'<\mathtt{Ro}(S)}} [\![M_{T\triangleright S}(u)\le u'<v'\le M_{T\triangleright S}(v)]\!]\Big) - |E(S)|+1\\
&= \Big(\sum_{\substack{\{u,v\}\in E(T)\\ u<v}} pl_S(M_{T\triangleright S}(u), M_{T\triangleright S}(v))\Big) - |E(S)|+1\\
&= DC(T,S) - |E(S)|+1
\end{aligned}$$

B Proof of Lemma 2

Proof. Since $A,B\subseteq \overline{X}$ we know that $A,B\subseteq \mathtt{Le}(S')$. In addition, since S' is a subtree of S, we know that $lca_S(A)=lca_{S'}(A)$ and similarly $lca_S(B)=lca_{S'}(B)$. Again, since S' is a subtree of S, any path in S' is also a path in S. In particular, the path from $lca_{S'}(A)$ to $lca_{S'}(B)$ in S' is also a path in S. Therefore we have $pl_S(lca_S(A), lca_S(B)) = pl_{S'}(lca_{S'}(A), lca_{S'}(B))$, and hence $pl_S(A,B)=pl_{S'}(A,B)$.

C Proof of Lemma 3

Proof. Let $v\in V(S')$ where $R=\Gamma(S,X,v)$. Node v must exist because $X\neq\emptyset$ and so S' has at least one node with degree two. Note that R is obtained from S' by the regrouping operation. Since $A,B\subseteq\overline{X}$, Lemma 2 applies on S and S', so we have $pl_S(A,B)=pl_{S'}(A,B)$.

We now consider the effect of the regrouping operation on the path lengths. First, (R1) does not modify any nodes or edges in the tree S', with the exception of v by introducing a new edge. However this new edge does not lie on any path in S', thus any path in S' remains unchanged after (R1). Further, (R2) may suppress nodes from S', hence reducing the length of a path in S'. This implies that

$pl_{S'}(A,B)\ge pl_R(A,B)$.

Overall, we have $pl_S(A,B)=pl_{S'}(A,B)\ge pl_R(A,B)$.

D Proof of Lemma 4

Proof. Since $A, B \subseteq \overline{\overline{X}}$, Lemma 2 applies on S with $\overline{X}$, where S' now becomes $S(\overline{\overline{X}}) = S(X)$. Therefore we have $pl_S(A, B) = pl_{S(X)}(A, B)$. Further, by (R1) we know that $S|X$ is a subtree of R, so we have $pl_{S|X}(A, B) = pl_R(A, B)$ because $A, B \subseteq X$. Lastly by the definition of restriction, $S|X$ is obtained from $S(X)$ by suppressing some nodes, therefore a path in $S(X)$ can only be made shorter in $S|X$, and so we have $pl_{S(X)}(A, B) \geq pl_{S|X}(A, B)$.

Overall, we have $pl_S(A, B) = pl_{S(X)}(A, B) \geq pl_{S|X}(A, B) = pl_R(A, B)$.

References

1. Maddison WP: **Gene Trees in Species Trees**. *Systematic Biology* 1997, **46**(3):523–536.
